# Supplementary material for: CLASS2: accurate and efficient splice variant annotation from RNA-seq reads
Source: Nucleic Acids Res. 2016 Mar 14;44(10):e98. doi: 10.1093/nar/gkw158 (PMC4889935; doi:10.1093/nar/gkw158)
Supplement: Supplementary Data [file gkw158_Supplementary_Data.zip › nar-00279-met-n-2016-File008.docx]

SUPPLEMENTARY MATERIAL FOR THE PAPER:

CLASS2: ACCURATE AND EFFICIENT SPLICE VARIANT ANNOTATION FROM RNA-SEQ READS

L. Song, S. Sabunciyan and L. Florea

**Supplementary Methods**

SM1. Performance comparison of CLASS (v.1.0.3) and CLASS2 (v.2.1.2)

SM2. Illustration of linear programming formulation for exon finding

SM3. Illustration of dynamic programming algorithm for transcript selection

SM4. Library preparation and sequencing

SM5. Primer sequences for PCR validation

**Supplementary Tables and Figures**

S1. Programs performance on simulated data (using sampled transcripts only as reference)

S2. Programs performance on simulated data (using GENCODE as reference)

S3. Performance inflation

S4. Correlation of predicted and sampled (‘truth’) expression values for transcripts

S5. Comparison of CLASS2 and Cufflinks (alternative splicing events)

S6. Performance of programs on the lymphocyte data sets (all transcripts vs multi-exon transcripts only)

S7. Programs’ performance on real data (Peripheral Blood Lymphocytes (PBL), polyA+ sample library)

S8. Programs’ performance on real data (Peripheral Blood Lymphocytes (PBL), rRNA-depleted library)

S9. Illustration of program output at the UBR4-CAPZB gene locus (Peripheral Blood Lymphocytes (PBL), rRNA-depleted sample)

S10. Program outcome on very deep sequencing data (IMR90, cytosol and nucleus)

S11. Program performance on very deep sequencing data (IMR90, cytosol and nucleus)

S12. Running times of programs

S13. Annotation of a newly sequenced organism (peach)

S14. Extending the peach gene annotation using RNA-seq

**SUPPLEMENTARY METHODS**

**SM1. Performance comparison of CLASS** (v.1.0.3; Song and Florea, *RECOMB-SEQ 2013*; *BMC Bioinformatics* 2013) **and CLASS2** (v.2.1.2). Performance was measured at exon, intron and full-transcript levels on the simulated data set. Recall = TP/(TP+FN), Precision = TP/(TP+FP), F-value = 2*Recall*Precision/(Recall+Precision). CLASS2_F0.01 denotes CLASS2 run in sensitive mode (‘-F 0.01’).

| Program | Predictions | Recall | Precision | F-value |
| --- | --- | --- | --- | --- |
| Transcript-level (reference: 22,544 transcripts) | | | | |
| CLASS_0_ | 15,076 | 0.439 | 0.656 | 0.526 |
| CLASS2 | 16,797 | 0.505 | 0.676 | 0.578 |
| CLASS2_F0.01 | 18,970 | 0.527 | 0.624 | 0.571 |
| Exon-level (reference: 118,624 exons) | | | | |
| CLASS_0_ | 84,925 | 0.702 | 0.939 | 0.803 |
| CLASS2 | 85,395 | 0.706 | 0.939 | 0.806 |
| CLASS2_F0.01 | 87,903 | 0.722 | 0.934 | 0.815 |
| Intron-level (reference: 95,133 introns) | | | | |
| CLASS_0_ | 72,172 | 0.742 | 0.979 | 0.845 |
| CLASS2 | 71,284 | 0.735 | 0.981 | 0.841 |
| CLASS2_F0.01 | 73,503 | 0.755 | 0.978 | 0.852 |

**SM2. Illustration of linear programming procedure for exon finding**

The example illustrates the linear program for the region R below (see also **Figure 1A**), divided by splice junction boundaries into five intervals R=*a.b.c.d.e* , and the set of exons {1,2,3,4}: X_1_ = (1,0,0,0,0), X_2_ = (0,0,1,0,0), X_3_ = (0,0,0,0,1) and X_4_ = (1,1,1,1,1). The set is feasible, because it explains all splice junctions. Let c_i,j_ be the coverage level for subexon i,j and C_j_ be the observed coverage of interval j, as defined in the text. The linear program is formulated as:

*(i) Additivity:*


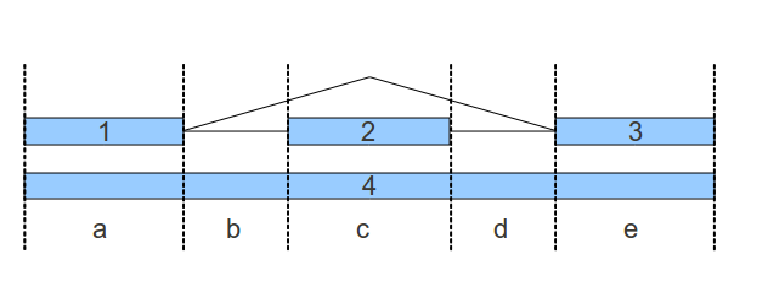
| c_1,a_+c_4,a_ –C_a_ | ≤ ε_a_

| c_4,b_ – C_b_ | ≤ ε_b_

| c_2,c_+c_4,c_-C_c_ | ≤ ε_c_

| c_4,d_-C_d_ | ≤ ε_d_

| c_3,e_+c_4,e_-C_e_ | ≤ ε_e_

*ii) Continuity:*

| c_4,a_-c_4,b_ | ≤ ε_4,a_

| c_4,b_-c_4,c_ | ≤ ε_4,b_

| c_4,c_-c_4,d_ | ≤ ε_4,c_

*iii) Conservation:*

| (c_1,a_+c_4,a_) L_a_ + c_4,b_ L_b_ + (c_2,c_+c_4,c_) L_c_ + c_4,d_ L_d_ + (c_3,e_+c_4,e_) L_e_ – (C_a_L_a_+C_b_L_b_+C_c_L_c_+C_d_L_d_+C_e_L_e_) | ≤ ε

*iv) Non-negativity:*

c_1,a_≥1, c_4,a_≥1, c_4,b_≥1, c_2,c_≥1, c_4,c_≥1, c_4,d_≥1, c_3,e_≥1, c_5,e_≥1

Optimization function: min ( Σ_j∈{a-e}_ε_j_ + Σ_i=1,4; j∈{a-e}_ε_i,j_ +ε )

**SM3. Illustration of dynamic programming algorithm for transcript selection**

Dynamic programming representation of transcript selection algorithm: Starting from a 5’ end, the algorithm calculates for each subpath L the maximum number of constraints for transcripts starting with L and ending at a 3’ end: f(L) = max { f(L’) + c(L,L’) }, where L’ is the minimum subpath such that all constraints starting in L cannot end past L’ (i.e., L’ is used as ‘look-ahead’). On the figure, c1 and c2 are constraints compatible with and used in the calculation of f(L). c3, c4 and c5 are not used in this stage, however, c3 and c4 had been used in the calculation of f(L’).an

**SM4. Library preparation and sequencing**

For analyses on real data, strand specific RNA-seq libraries were constructed for the lymphocyte samples using the TruSeq RNA SamplePrep Guide version 15008136_A with modifications. Briefly, for the poly A preparation mRNA was purified from 2 µg of total RNA using Illumina RNA purification beads, the resulting mRNA was fragmented using the Illumina Elute, Prime, Fragment Mix and 1^st^ strand cDNA was synthesized following the TruSeq RNA protocol. Second strand cDNA was synthesized using 8 µl of 10X NEBNext® Second Strand Synthesis (dNTP-Free) Reaction Buffer, 2µl of 10X SuperScript II RT Buffer (NEB), 250uM of each dATP, dUTP, dCTP and dGTP, all of the material from the 1^st^ strand cDNA reaction and 4µl of second strand enzyme (NEB) in a total of 100µl. The reaction was incubated at 16^0^ C for 2.5 hours. The resulting double stranded cDNA was purified, end repaired and adenylated following the TruSeq RNA Sample Prep protocol. One microliter of Illumina adapters were used for the ligation following the TruSeq RNA Sample Prep protocol. The adapter ligated cDNA library was then purified using Ampure beads and subjected to USER enzyme digestion in 5 µl of 10X HotStar PCR buffer (Qiagen), 1 unit of USER enzyme (NEB) in a total of 50µl. This reaction was incubated at 37^0^ C for 15 minutes and the enzyme was inactivated by heating to 95^0^ C for 5 minutes. The digested cDNA was purified using Ampure beads and PCR amplified following the protocol in the TruSeq RNA Sample Prep protocol. For the rRNA-depleted library we started with 5µg of total RNA and removed the rRNA using the Ribo-zero magnetic gold kit (Epicenter) instead of purifying mRNA. Otherwise, the library preparation protocol was identical to the procedure described above.

**SM5. Primer sequences for PCR validation**

| PCR | Primer Name | Sequence |
| --- | --- | --- |
| CACNA2D4-1 | CACNA2D4_A1For_94 | GGGTCCTGCTTCTTGTGTTT |
|  | CACNA2D4_A1Rev_336 | GACAGTGGGGATAGGTGACC |
| CACNA2D4-2 | CACNA2D4_A2For_57 | CACGAACACGGGGTACTCC |
|  | CACNA2D4_A2Rev_175 | TGCCACCATGTTTTCCTGTG |
| KLRF1-1 | KLRF1_A1For_165 | GGAGTTCTGCCCAAACATCTC |
|  | KLRF1_A1Rev_354 | ACTGTGGAGTGTACTAATAGAGC |
| KLRF1-2 | KLRF1_A2For_172 | TGCCCAAACATCTCAACTTACA |
|  | KLRF1_A2Rev_421 | CCGTATTAGACTGTATGCCACT |

**SUPPLEMENTARY FIGURES AND TABLES**

**Figure S1.** Performance of programs on simulated data. Accuracy was measured at exon (X), intron (I) and full transcript (T) levels, by comparison to the subset of reference transcripts sampled by FluxSimulator. Recall = TP/(TP+FN), Precision = TP/(TP+FP) and F = 2*Recall*Precision/(Recall+Precision).

**Figure S2.** Performance evaluation of programs on simulated data, when measured against the full set of GENCODE gene annotations (compare to measurements against the subset of simulated transcripts only, **Fig. 2** in the main text and **Supplementary Figure S1**). Recall = TP/(TP+FN), Precision = TP/(TP+FP) and F-value = 2 x Recall x Precision/(Recall + Precision) were measured at the exon (top), intron (center) and full-transcript (bottom) levels.


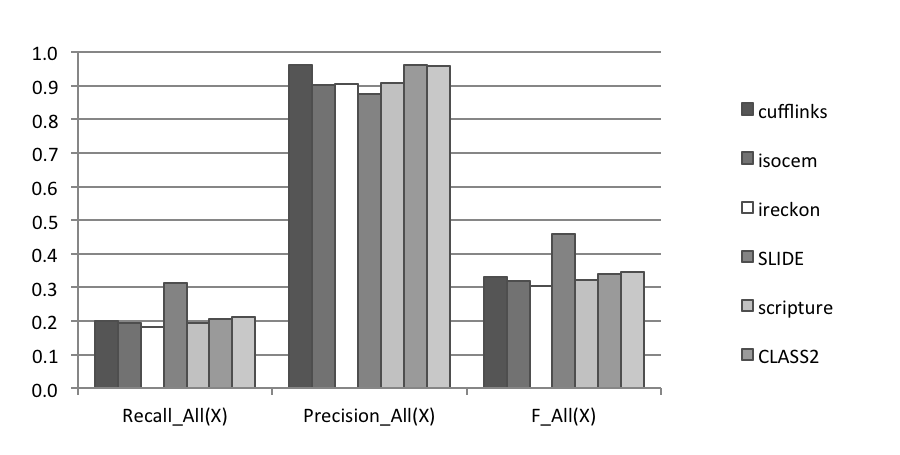

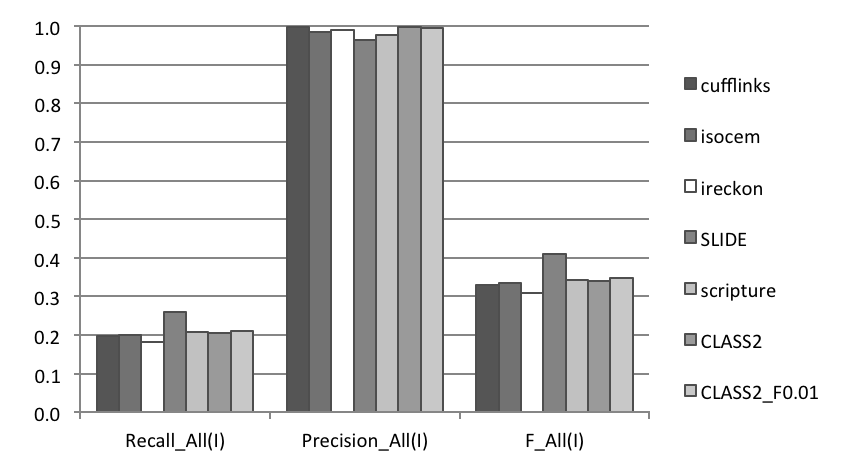

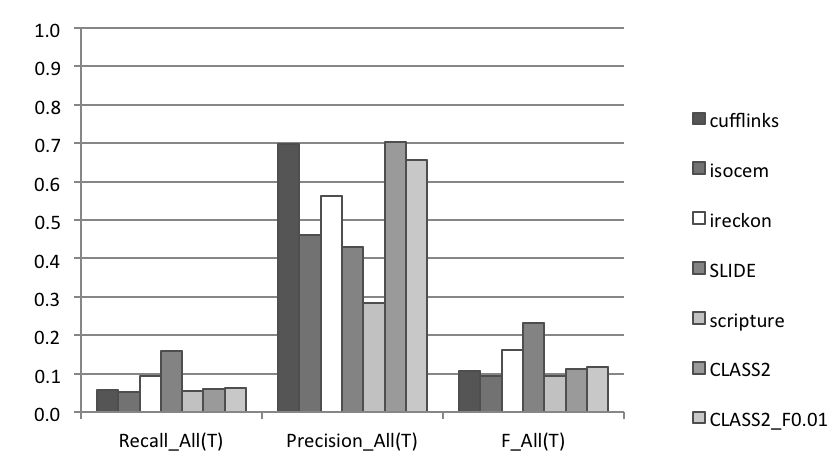


**Table S3. Performance inflation.** Sources of program performance inflation, when compared against the entire set of GENCODE gene annotations, as measured by percentage increase *(PCI) = 100 *[(Match_GENCODE/Match_sim) -1].* *Match_GENCODE* = number of reference transcripts reconstructed by the program from the entire GENCODE annotation set, and *MATCH_sim* = number of such transcripts in the subset simulated by FluxSimulator (‘ground truth’). PCI values were calculated separately for all transcripts (column 2) and for multi-exon transcripts only (column 5, *‘Nx’*).

| Program | All Txpts | PCI all | >1 exon txpts | PCI  Nx | Single-exon | | Multi-exon | |
| --- | --- | --- | --- | --- | --- | --- | --- | --- |
|  |  |  |  |  | Variant | Paralog | Variant | Paralog |
| CLASS2 | 632 | 5.6 | 385 | 3.8 | 154 | 93 | 353 | 47 |
| CLASS2_F0.01 | 801 | 6.8 | 554 | 5.2 | 154 | 93 | 519 | 50 |
| Cufflinks | 678 | 6.3 | 313 | 3.4 | 218 | 147 | 289 | 36 |
| Cufflinks_F0.01 | 1118 | 9.8 | 733 | 7.5 | 226 | 159 | 690 | 55 |
| IsoCEM | 1742 | 20.2 | 825 | 11.9 | 222 | 694 | 701 | 133 |
| Scripture | 2012 | 22.4 | 1706 | 22.2 | 176 | 130 | 1620 | 98 |
| iReckon | 7328 | 64.4 | 2398 | 25.1 | 484 | 4445 | 2011 | 397 |
| SLIDE | 17163 | 120.7 | 15825 | 128.1 | 534 | 783 | 15182 | 656 |

**Figure S4. Correlation of predicted and sampled (‘truth’) expression values for transcripts.** (A) Scatterplot of FluxSimulator-generated expression levels (‘truth’) and abundance values estimated by CLASS2. (B) Correlations between sampled expression levels and abundance estimates by each of the tested programs. Fully-reconstructed transcripts by each program, which could be unambiguously associated with sampled (reference) transcripts were included. R2 = Pearson correlation.

(A) (B)

| Program | R^2^ |
| --- | --- |
| cufflinks | 0.804 |
| isoCEM | 0.977 |
| iReckon | 0.708 |
| SLIDE | 0.878 |
| Scripture | 0.674 |
| CLASS2 | 0.972 |
| CLASS2_F0.01 | 0.972 |

**Figure S5. Comparison of CLASS2 and Cufflinks in detecting alternative splicing events.** Recall (R, horizontal axis) and precision (P, vertical axis) of CLASS2 (light grey) and Cufflinks (dark grey) are shown for various stringency settings (‘-F f’, with f=0.01, 0.02, 0.03, 0.05, 0.1 and 0.2; right-to-left in the plots). The parameter ‘F’ controls the expression range of isoforms reported, as a fraction of the expression level of the most abundant isoform for the gene. Both programs have high specificity for stringent settings, but CLASS2 can detect more events overall and, for the same sensitivity (recall) level, has significantly higher precision than Cufflinks.

**Table S6. Performance of programs on the lymphocyte RNA-seq data sets (polyA-selected, top; rRNA-depleted, bottom)**, when considering all transcripts of a gene and multi-exon transcripts only, respectively. R = recall, P = precision, F = 2 * R * P /(R+P).

| All (polyA+) | | | | | Multi-exon only (polyA+) | | | |
| --- | --- | --- | --- | --- | --- | --- | --- | --- |
| Program | Transcripts | R | P | F | Transcripts | R | P | F |
| GENCODE | 198188 | - | - | - | 172004 | - | - | - |
| CLASS2 | 42185 | 0.071 | 0.331 | 0.117 | 35502 | 0.079 | 0.385 | 0.132 |
| CLASS2_F0.01 | 58444 | 0.081 | 0.271 | 0.125 | 51761 | 0.090 | 0.301 | 0.139 |
| Cufflinks | 92956 | 0.065 | 0.139 | 0.089 | 27283 | 0.063 | 0.394 | 0.108 |
| isoCEM | 367524 | 0.057 | 0.032 | 0.041 | 27163 | 0.044 | 0.280 | 0.076 |
| iReckon | 60347 | 0.130 | 0.392 | 0.195 | 40809 | 0.093 | 0.390 | 0.150 |
| Scripture | 373656 | 0.074 | 0.039 | 0.051 | 337126 | 0.076 | 0.039 | 0.051 |
| All (rRNA-) | | | | | Multi-exon only (rRNA-) | | | |
| CLASS2 | 38156 | 0.035 | 0.178 | 0.058 | 29072 | 0.037 | 0.220 | 0.064 |
| CLASS2_F0.01 | 42218 | 0.038 | 0.174 | 0.062 | 33134 | 0.040 | 0.210 | 0.068 |
| Cufflinks | 1139440 | 0.116 | 0.020 | 0.035 | 18410 | 0.015 | 0.144 | 0.028 |
| isoCEM | 410743 | 0.039 | 0.017 | 0.023 | 7835 | 0.006 | 0.126 | 0.011 |
| iReckon | 71069 | 0.154 | 0.399 | 0.222 | 31979 | 0.065 | 0.350 | 0.110 |
| Scripture | 443844 | 0.084 | 0.037 | 0.052 | 105278 | 0.043 | 0.071 | 0.054 |

**Figure S7. Performance evaluation of programs on real data (lymphocyte, polyA+ sample).** Predicted transcripts were compared to the GENCODE annotations for evaluation. Recall, Precision and F-value (see definition above) were measured and shown at exon (X), intron (I) and transcript (T) level (first three panels). Last panel: estimated relative performance compared to Cufflinks after accounting for inflation. The values for Cufflinks (reference) are 0.


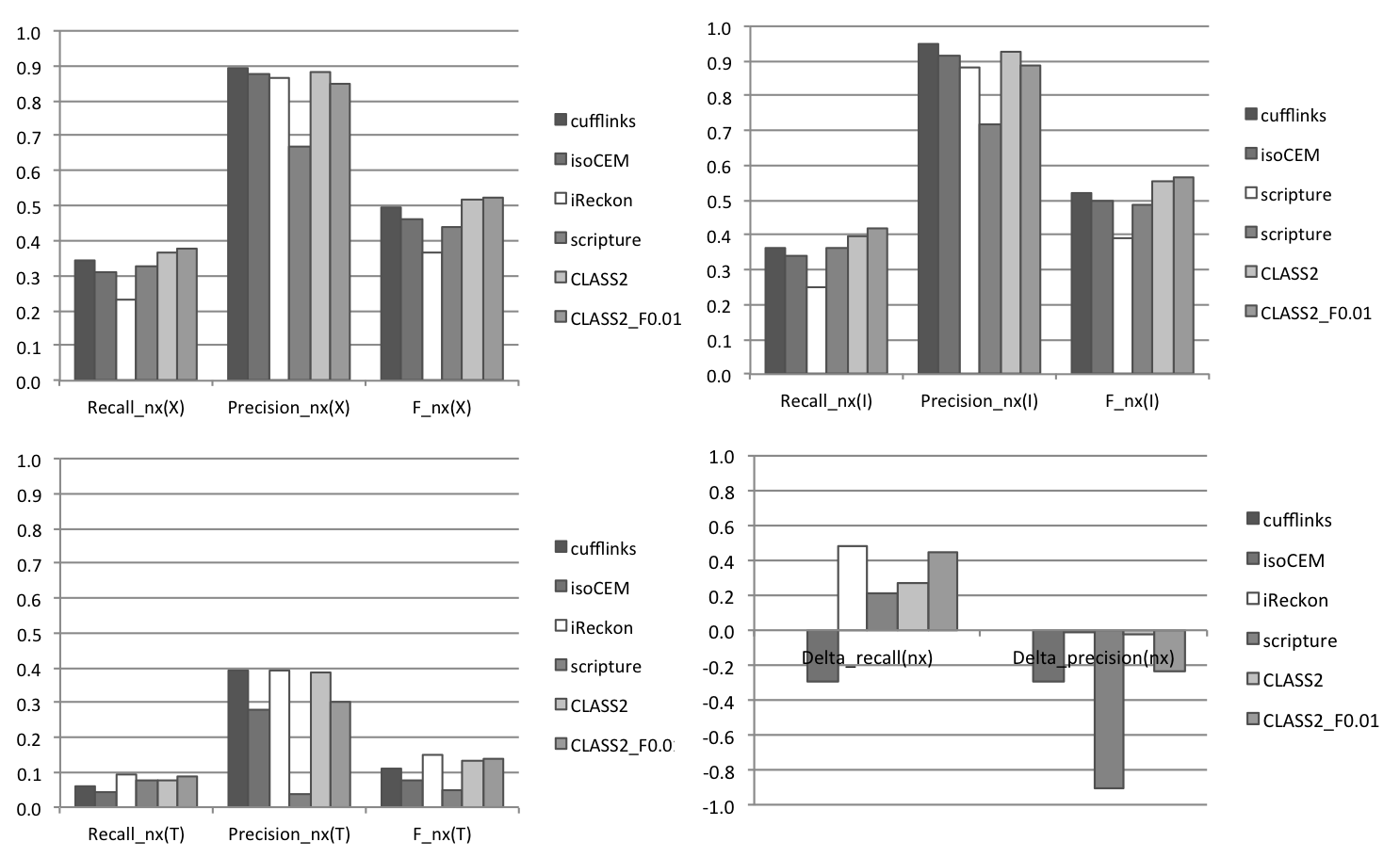


**Figure S8. Performance evaluation of six programs on real data (lymphocyte, rRNA-depleted sample).** Predicted transcripts were compared to the GENCODE annotations for evaluation. Recall, Precision and F-value (see definition above) were measured and shown at exon, intron and transcript level (first three panels). Last panel: estimated relative performance compared to Cufflinks after accounting for inflation. The values for Cufflinks (reference) are 0.


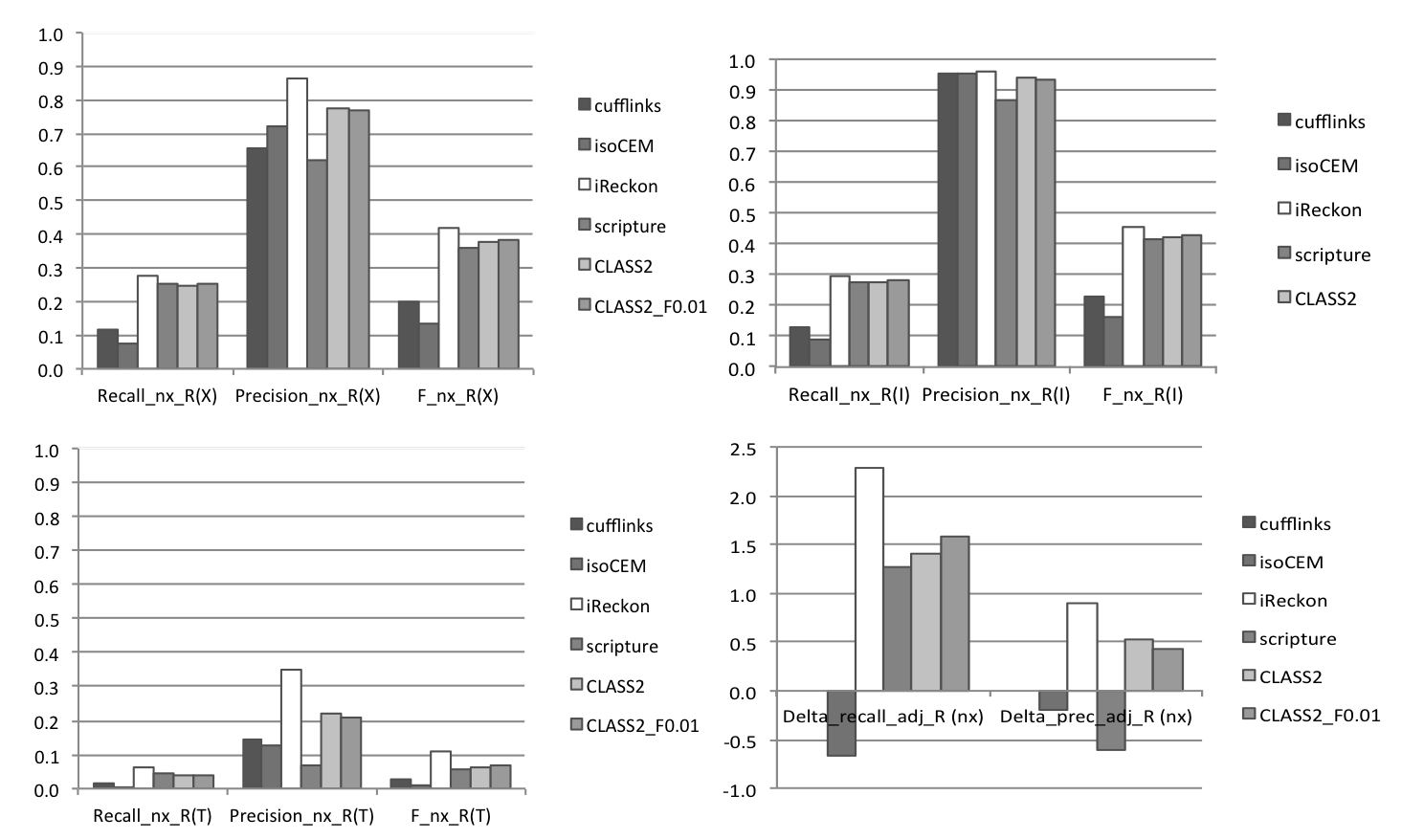


**Figure S9**. **Illustration of program output at the UBR4-CAPZB gene locus** (lymphocyte, rRNA-depleted sample). IsoCEM and Cufflinks fail to identify full-length transcripts models, and are confounded by intronic noise (red circle).


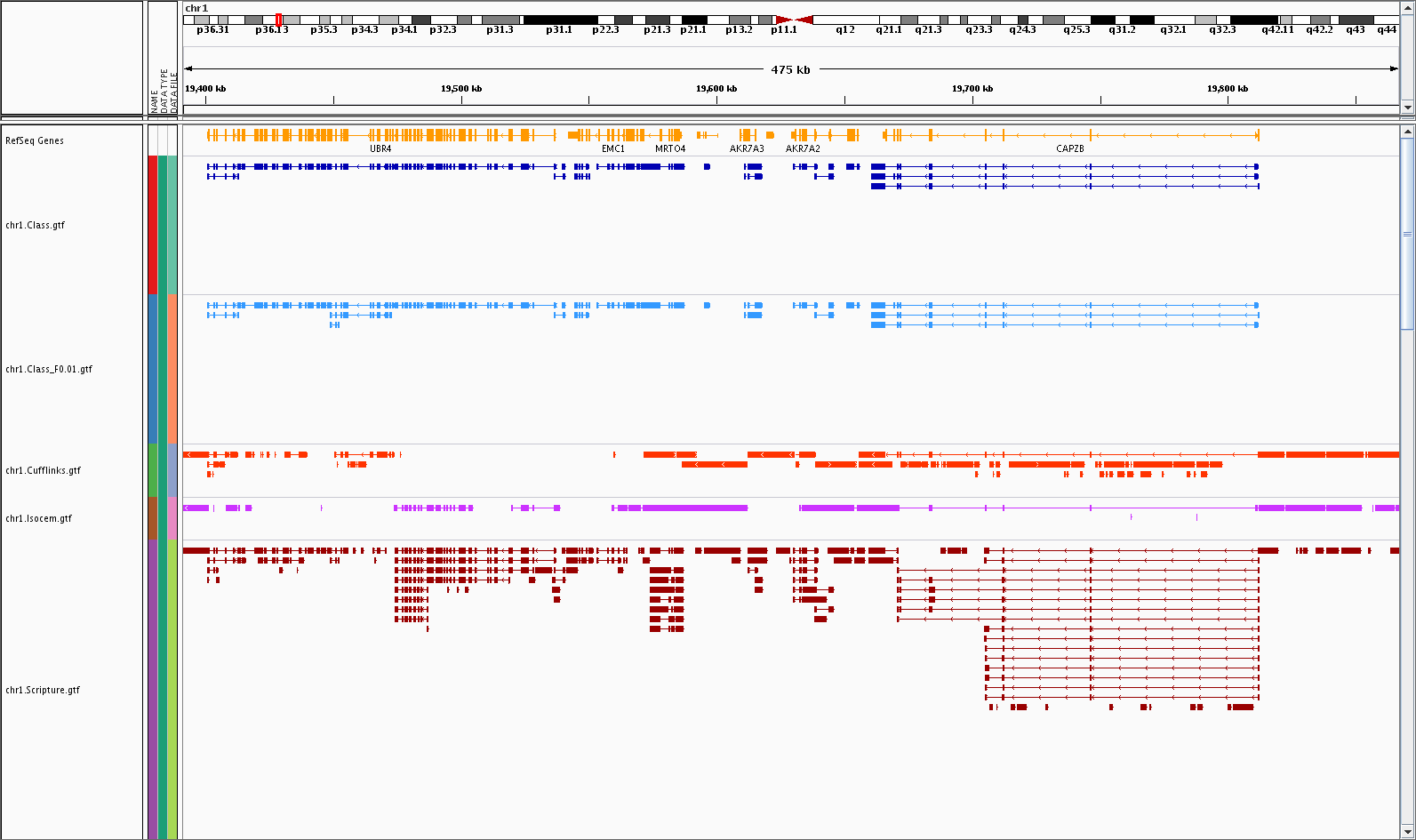


GENCODE

Cufflinks

CLASS

IsoCEM

Scripture

CLASS_F0.01

**Table S10. Performance of programs on very deep sequencing data sets (IMR90 cellular fractions: cytosol and nucleus),** when considering only multi-exon transcripts. *Match_ref* = # reference transcripts that match the predictions, *Match_pred* = # predicted transcripts that match the reference, R = recall, P = precision, F = F-value = 2 * R * P /(R+P). Results are shown for chromosome 1 only.

| Cytosol | | | | | | |
| --- | --- | --- | --- | --- | --- | --- |
| Program | Transcripts | Match_ref | Match_pred | R | P | F |
| GENCODE | 15493 | - | - | - | - | - |
| CLASS2 | 3029 | 1053 | 1053 | 0.068 | 0.348 | 0.114 |
| CLASS2_F0.01 | 3836 | 1183 | 1183 | 0.076 | 0.308 | 0.122 |
| Cufflinks | 2508 | 621 | 621 | 0.040 | 0.248 | 0.069 |
| isoCEM | 2479 | 722 | 722 | 0.047 | 0.291 | 0.080 |
| iReckon | 4512 | 1730 | 1730 | 0.112 | 0.383 | 0.173 |
| Scripture | 14621 | 971 | 971 | 0.063 | 0.066 | 0.064 |
| Nucleus | | | | | | |
| CLASS2 | 6084 | 992 | 992 | 0.064 | 0.163 | 0.092 |
| CLASS2_F0.01 | 10216 | 1141 | 1141 | 0.074 | 0.112 | 0.089 |
| Cufflinks | 2714 | 561 | 561 | 0.036 | 0.207 | 0.062 |
| isoCEM | 2236 | 277 | 277 | 0.018 | 0.124 | 0.031 |
| iReckon | 5769 | 1539 | 1539 | 0.099 | 0.267 | 0.145 |
| Scripture | 45247 | 764 | 764 | 0.049 | 0.017 | 0.025 |

**Figure S11. Program performance on very deep sequencing data (IMR90, cytosol – top and nucleus - bottom)**, measured against the full set of GENCODE annotations (chromosome 1 only). Recall, ‘precision’ and F-value are as defined above.

**(A)**

**(B)**

**Table S12. Running times of transcript assembly algorithms** for the data sets used in the evaluation. Run times for CLASS2 and CLASS2_F0.01 are largely the same, since the algorithm first detects a comprehensive set of transcripts and then applies the expression cutoff to select a subset. Run times for iReckon included the time for internally re-mapping the reads to the genome with bwa, and were excluded. All times measured on a Unix machine with 512 GB RAM and 2100 MHz CPU, single-threaded. Memory usage for CLASS2 for all tasks was <3 GB RAM. PBL = Peripheral Blood Lymphocytes.

| Program/  Data set | Time (wall clock) | | | | |
| --- | --- | --- | --- | --- | --- |
|  | Sim | PBL  polyA+ | PBL  rRNA- | IMR90  Cytosol  (chr1) | IMR90  Nucleus  (chr1) |
| CLASS2 | 390m | 296m | 244m | 31m | 747m |
| Cufflinks | 488m | 1679m | 4245m | 84m | 548m |
| isoCEM | 168m | 169m | 225m | 18m | 67m |
| iReckon | 6097m | 5696m | 17481m | Na | Na |
| SLIDE | ~1.5 weeks | Na | Na | Na | Na |
| Scripture | 1039m | 1262m | 1574m | 27m | 75m |

**Table S13. Annotation of a newly sequenced organism (peach).** Summary of mapping and assembly results are shown for the four RNA-seq samples (SRR531862 – embryos and cotyledons, SRR531863 – root, SRR531864 – fruit and SRR531865 – leaf). ‘Mapped’ represents the number of reads mapped with 10 or fewer matches on the peach genome. The last two columns give the numbers of loci (‘genes’) and transcripts assembled with CLASS2, using the default and the sensitive (‘-F 0.01’) settings.

| **Set** | **Reads** | **Mapped** | **Genes**  **(CLASS2)** | **Transcripts**  **(CLASS2)** | **Genes**  **(CLASS2_F0.01)** | **Transcripts**  **(CLASS2_F0.01)** |
| --- | --- | --- | --- | --- | --- | --- |
| SRR531862 | 42394368 | 35846028 | 17320 | 22617 | 17,322 | 27,442 |
| SRR531863 | 41589898 | 29261488 | 16313 | 20799 | 16,313 | 24,614 |
| SRR531864 | 42341754 | 25016427 | 16320 | 18,397 | 16,321 | 19,816 |
| SRR531865 | 38883238 | 24707106 | 12083 | 13,752 | 12,083 | 14,935 |

**Table S14. Extending the annotation of the peach genome.** Known and novel loci and transcript variants across the four samples were discovered by comparison to the existing reference annotations (28,702 transcripts; 27,864 genes), using cuffcompare. Numbers in parentheses indicate variants present in two or more of the four samples.

| **Program** | **Known loci** | | | **Novel loci** |
| --- | --- | --- | --- | --- |
|  | **Full** | **Contained** | **Variants** |  |
| CLASS2 | 9148 (7022) | 10248 (1149) | 18494 (4765) | 969 (969) |
| CLASS2_F0.01 | 9236 (7079) | 10256 (1157) | 27161 (5952) | 1040 (1040) |
