# Supplementary material for: CLASS2: accurate and efficient splice variant annotation from RNA-seq reads
Source: Nucleic Acids Res. 2016 Mar 14;44(10):e98. doi: 10.1093/nar/gkw158 (PMC4889935; doi:10.1093/nar/gkw158)
Supplement: SUPPLEMENTARY DATA [file supp_44_10_e98__index.html]

CLASS2: accurate and efficient splice variant annotation from RNA-seq reads — CLASS2: accurate and efficient splice variant annotation from RNA-seq reads — SUPPLEMENTARY DATA 

# CLASS2: accurate and efficient splice variant annotation from RNA-seq reads

## SUPPLEMENTARY DATA

- SUPPLEMENTARY DATA
- SUPPLEMENTARY DATA
